# Evaluation of the Multimycotoxin-Degrading Efficiency of *Rhodococcus erythropolis* NI1 Strain with the Three-Step Zebrafish Microinjection Method

**DOI:** 10.3390/ijms22020724

**Published:** 2021-01-13

**Authors:** Edina Garai, Anita Risa, Emese Varga, Mátyás Cserháti, Balázs Kriszt, Béla Urbányi, Zsolt Csenki

**Affiliations:** 1 Department of Aquaculture, Institute for Conservation of Natural Resources, Faculty of Agricultural and Environmental Sciences, Szent István University, H-2100 Gödöllő, Hungary; edina.garai@phd.uni-szie.hu (E.G.); urbanyi.bela@szie.hu (B.U.); 2 Institute of Aquaculture and Environmental Safety, Hungarian University of Agriculture and Life Sciences, H-2100 Gödöllő, Hungary; ranita513@gmail.com (A.R.); cserhati.matyas@szie.hu (M.C.); kriszt.balazs@szie.hu (B.K.); 3 Department of Environmental Safety and Ecotoxicology, Institute for Conservation of Natural Resources, Faculty of Agricultural and Environmental Sciences, Szent István University, H-2100 Gödöllő, Hungary; 4 Department of Applied Chemistry, Faculty of Food Science, Szent István University, H-1118 Budapest, Hungary; varga.emese@etk.szie.hu

**Keywords:** aflatoxin B_1_, T-2, zearalenone, combined exposure, combined toxicity

## Abstract

The multimycotoxin-degrading efficiency of the *Rhodococcus erythropolis* NI1 strain was investigated with a previously developed three-step method. NI1 bacterial metabolites, single and combined mycotoxins and their NI1 degradation products, were injected into one cell stage zebrafish embryos in the same doses. Toxic and interaction effects were supplemented with UHPLC-MS/MS measurement of toxin concentrations. Results showed that the NI1 strain was able to degrade mycotoxins and their mixtures in different proportions, where a higher ratio of mycotoxins were reduced in combination than single ones. The NI1 strain reduced the toxic effects of mycotoxins and mixtures, except for the AFB1+T-2 mixture. Degradation products of the AFB1+T-2 mixture by the NI1 strain were more toxic than the initial AFB1+T-2 mixture, while the analytical results showed very high degradation, which means that the NI1 strain degraded this mixture to toxic degradation products. The NI1 strain was able to detoxify the AFB1, ZEN, T-2 toxins and mixtures (except for AFB1+T-2 mixture) during the degradation experiments, which means that the NI1 strain degraded these to non-toxic degradation products. The results demonstrate that single exposures of mycotoxins were very toxic. The combined exposure of mycotoxins had synergistic effects, except for ZEN+T-2 and AFB1+ZEN +T-2, whose mixtures had very strong antagonistic effects.

## 1. Introduction

Mycotoxins are secondary metabolites of fungi, which are common pollutants of global food and feed chains. Change in global climate has increased the size of the areas suitable for fungal growth and cause considerable economic loss by infection and mycotoxins produced [1,2,3,4]. The major mycotoxin-producing fungi genera are *Aspergillus*, *Penicillium*, and *Fusarium*, which produce most mycotoxins with different toxic effects such as hepatotoxicity, reproductive toxicity, nephrotoxicity, carcinogenicity, and immunotoxicity [3,5,6,7]. Mycotoxins that mean considerable potential risk to human and animal health are aflatoxins, trichothecenes, zearalenone, ochratoxin A, fumonisins, and ergot alkaloids [3].

*Aspergillus flavus* and *A. parasiticus* fungi species produce aflatoxin B_1_ (AFB1) and mainly infect peanuts, corn, wheat, rice, cottonseed, copra, nuts, and various foods. AFB1 has a carcinogenic effect and causes various diseases in animals and humans [3,5,7,8]. Acute toxicological effects are liver damage, decreased egg- and milk production, feed refusal, decreased growth, anemia, jaundice, weight loss, anorexia, hemorrhage, embryotoxicity, and carcinoma in rainbow trout, pig, cattle, poultry, duckling, turkeys, and chickens [8,9,10,11,12,13]. Chronic effects are hepatocellular carcinoma, lower reproductivity, decreased cellulose digestion, volatile fatty acid formation, and proteolysis in cattle, swine, and rainbow trout [9,14]. The effects on humans include liver cancer, chronic hepatitis C infection, and Reye’s syndrome (encephalopathy and visceral degeneration in children). Incidence of these diseases have been recorded in India (1974) and Kenya (2004 and 2005) [3,5,7,15].

*Fusarium graminearum* and *F. culmorum* fungi species produce zearalenone (ZEN) and mainly infect corn, hay, and pelleted commercial feed. ZEN has an estrogenic effect in humans and animals [3,8,16]. In general, toxicological effects are swollen vulva, mammary gland enlargement, hyperestrogenism, feminization in young male animals (testicular atrophy, swollen prepuce), decreased libido, decreased spermatogenesis, infertility, and embryonic death in swine, cattle, poultry, and laboratory rodents [3,8,16]. The effects on humans are premature puberty, premature thelarche, prepubertal breast enlargement in boys, pseudopuberty in girls, and involves cervical cancer and lasting effects on the endocrine system [3,17,18,19]. These effects have been studied in Puerto Rico and Hungary [3,17,18,19]. 

*Fusarium poae* and *F. sporotrichioides* fungi species produce T-2 and mainly infect corn, wheat, commercial feed, and mixed feed. Acute toxicological effects are dermatitis, feed refusal, vomiting, testis and ovary aberrations, hemorrhages and necrosis of stomach, depressed growth, early life stage toxicity in cats, dairy cattle, dogs, pigs, ducklings, zebrafish, and rainbow trout [3,8,20,21,22,23,24]. Chronic exposure causes dizziness, excessive salivation, fatigue, secondary infections (pneumonia) and abdominal pain in chickens, mice, rats, and rhesus monkeys [25,26,27,28]. The effects on humans could be connected to alimentary toxic aleukia (ATA; sepsis, agranulocytosis, atrophy of the bone marrow, mortality), however, it is not clear whether T-2 causes the disease alone or with other mycotoxins [3,22,23,29,30,31]. Incidences of disease have been described in the USSR (Union of Soviet Socialist Republics; USSR) (1941–1947), China (1984–1985), and India (1987) [22,23,29,30,31]. 

Mycotoxins are produced by different fungus species and some fungi are able to produce different toxins [3,6,32,33]. The natural co-occurrence of mycotoxins increases the risk of exposure to several mycotoxins at the same time in humans and animals [32,34]. The toxicity of mycotoxin mixtures is not always possible to predict based on their individual effects as interactions among mycotoxins could be additive, antagonistic, or synergistic [6,32,34]. Earlier worldwide examination of mycotoxin levels in food and feed indicate that more than 70% [6,35] of samples were contaminated with at least one mycotoxin [6,35]; another global measurement showed that 48% [6,36] of samples were contaminated with at least two mycotoxins. In a previous study, the mycotoxin content of AFB1, ZEN, and T-2 were investigated in compound animal feed and more than 57% of samples were contaminated with three types of mycotoxins [7]. 

Increased health risk due to co-contamination of mycotoxins confirms the elimination of mycotoxins from the food and feed chain [37,38]. Several processes of removal and detoxification of mycotoxins have been investigated, however, most of these are ineffective, decrease nutritional values, or produce toxic derivates [31,37,38,39,40,41,42,43]. Biological transformation may be an ideal approach to decrease mycotoxins. Several previous studies have described bacterial degradation and detoxification of AFB1 [44,45], ZEN [46,47], and T-2 [37,48] with different strains, however, limited data are available about the degradation and detoxification of mycotoxin mixtures [49]. In an earlier study, Ery4 laccase from *Pleurotus eryngii* was used for the degradation of AFB1+ZEN and fumonisin B_1_ (FB1)+T-2 mixtures. AFB1+ZEN were degraded by 86% and 100%, while FB1+T-2 by 25% and 100% [50]. Rumen fluid was able to degrade 90% of ZEN and 100% of T-2 toxin, but had no effect on AFB1 [51]. Members of the genus *Rhodococcus* were used for degradation of AFB1+ZEN+T-2 mixtures, the *R. pyridinivorans* K408 strain was able to degrade AFB1+ZEN mixture by 99% and 96%, the *R. rhodochrous* NI2 strain degraded AFB1+T-2 by 99% and 97%, and the *R. erythropolis* NI1 strain degraded AFB1+ZEN+T-2 by 99% and 98% and 96% [52]. Degradation does not mean detoxification in every case, because mycotoxins can transform into more toxic metabolites (such as AFB1—AFB1-8,9-epoxide; ZEN—α-zearalenol; T-2—3-hydroxy-T-2) and bacterial metabolites can also be toxic (such as bacterial metabolites of the *Rhodococcus rhodochrous* NI2 strain) [49,53]. European Food Safety Authority (EFSA) guidelines suggest that the toxicity of degradation metabolites needs to be examined with *in vivo* toxicological methods [54,55]. 

Previously, the Csenki-Garai three-step method ((1) determination of mycotoxin toxicity baseline, (2) examination of bacterial metabolites toxicity, and (3) identification of degradation products toxicity) was developed, which is a microinjection-based technique in a zebrafish model system, for qualification of degradation and detoxification efficiency of bacteria, and suitable for indirect testing of toxin metabolites [53,56]. During the development of the method, it was demonstrated that the injection volumes alone do not cause mortality or other malformations in the treated embryos. In the case of a well optimized method, injection volume variations can be kept within ±20%, according to the OECD 236 (Organisation for Economic Cooperation and Development; OECD) test guideline’s recommendations and result reliability can be ensured [56]. Csenki et al. described *Cupriavidus basilensis* ŐR16 bacteria strain ochratoxin-A (OTA) degradation efficiency, which was used to developed a suitable microinjection test. The following results were used as the basis for the test: the ŐR16 strain could degrade almost 100% of OTA, the OTA major degradation metabolite was OTα, and neither of the samples (bacteria metabolites and degradation products) had any effect in the mice test, thus confirming that the zebrafish embryo—thanks to their sensitivity—proved to be a good model for this type of study. The results also showed that the effects observed in the treatments were derived only from the toxin and the normal and degradation metabolites of the microbe [56]. With the help of the Csenki-Garai three-step method, seven different bacterial strains and T-2 toxin-degrading properties were examined for classifying the strains [53]. The results confirmed that this microinjection technique may provide an opportunity for the selection of microbial strains that are able to degrade toxins and the identification of the most effective and environmentally safe microbes from the selected strains.

In this study, we investigated whether the Csenki-Garai three-step method is appropriate to evaluate the multimycotoxin-degrading efficiency of the *Rhodococcus erythropolis* NI1 strain. The objective of this experiment was to examine the toxic effects of AFB1, ZEN, and T-2 individually or in combination as well as that of their degradation products by the NI1 strain on zebrafish embryos. In addition, this study explored the interactions among these mycotoxins. 

## 2. Results and Discussion

Toxicological effects of *Rhodococcus erythropolis* NI1 bacterial metabolites, single and combined mycotoxins and their NI1 degradation products were examined with the same injected doses (0.22, 0.52, 1.77, and 4.17 nL). These injected volumes were selected so that the mortality values of toxins and mixtures were interpretable above and below the baseline in every dose [53]. The initial concentrations of AFB1, ZEN, and T-2 were 1 mg/L in individual solutions, and 1-1 mg/L in combinations (ratio was 1:1), where the initial concentration was in the range where interactions could be plotted (antagonism, additive, and synergism) for simultaneous exposure of mycotoxin mixtures.

### 2.1. Toxicity Effects of NI1 Bacterial Metabolites

**Figure 1:**
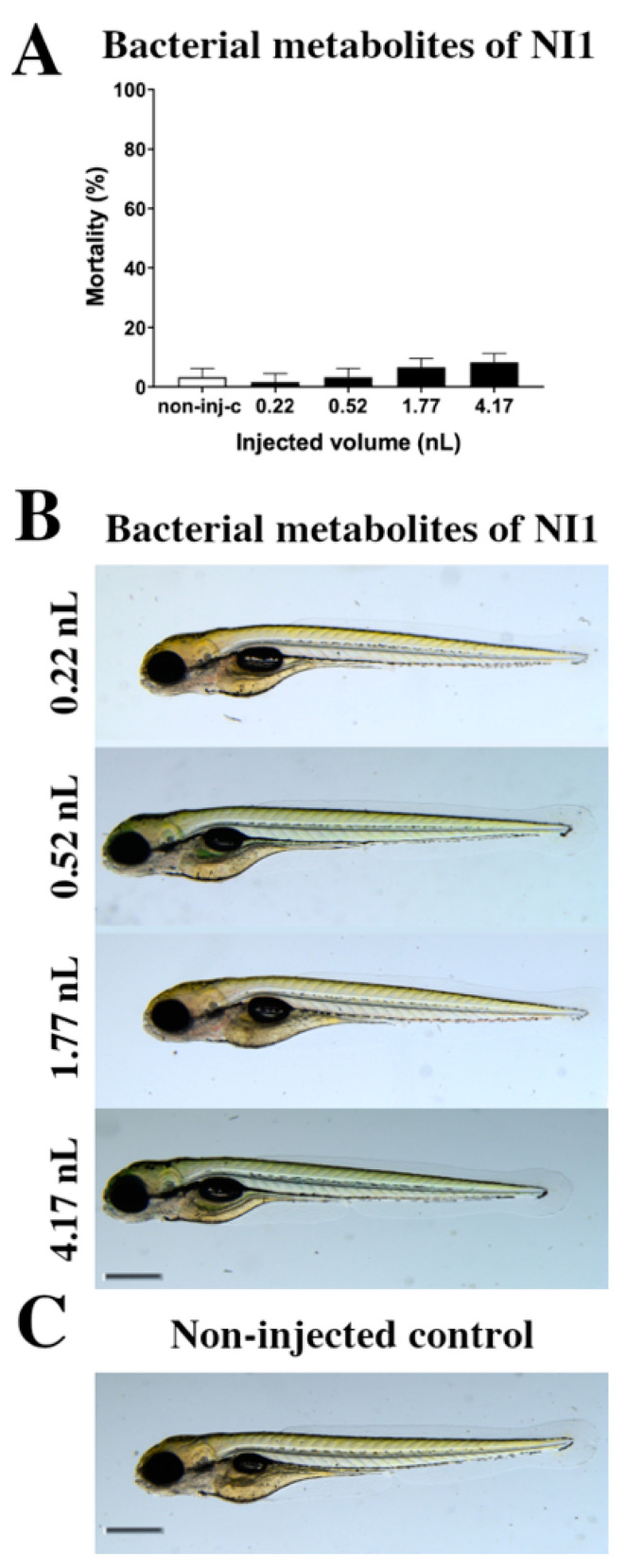
Effects of the *Rhodococcus erythropolis* NI1 strain bacterial metabolites on 120 hpf zebrafish embryos. Mortality results of NI1 strain bacterial metabolites (**A**) were checked following 120 h of injection. Lethality data are expressed as mean ± SD from three independent experiments in triplicate. Kruskal–Wallis followed by Dunn’s post-hoc test was used. Mortality values were compared to the non-injected control (non-inj-c). Larvae had no phenotypic lesions (**B**) at either dose after 120 h of injection. Scale bar: 1 mm. Phenotypes of treated groups were compared to the non-injected control groups (**C**). Quantitative data of developmental dysfunctions in zebrafish embryos are in the Supplementary Materials (Supplementary Table S1).

The toxicity effects of bacterial metabolites of the *Rhodococcus erythropolis* NI strain (Figure 1) were tested. Bacterial metabolites increased the mortality rate of 120 h post fertilization (hpf) embryos (Figure 1A), but significant differences were not observed compared to the non-injected control (non-inj-c). The mortality range was from 3% (±2.89%) to 10% (±5.00%) between 0.22 and 4.17 nL doses. Sublethal effects of bacterial metabolites (Figure 1B) were not observed at either injected dose on 120 hpf larvae, compared to the non-injected control (Figure 1C). These results are in good agreement with a previous study, which also suggests that bacterial metabolites of the *Rhodococcus erythropolis* NI strain had no effects (lethal and sublethal) on zebrafish embryos [53].

### 2.2. Toxicity Effects of (AFB1, ZEN, T-2 in Individual and in Combination, and Their NI1 Degradation Products on Zebrafish Embryos

#### 2.2.1. AFB1 Treatment

**Figure 2:**
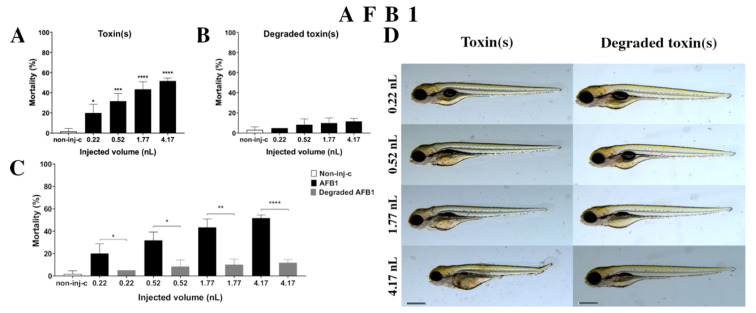
Effects of AFB1 (**A**) and its degradation products (**B**) by the *Rhodococcus erythropolis* NI1 strain on 120 hpf zebrafish embryos. Mortality data are expressed as mean ± SD from three independent experiments in triplicate. Kruskal–Wallis followed by Dunn’s post-hoc test and Mann–Whitney test were used. Mortality values were compared to the non-injected control (non-inj-c) (* *p* < 0.05, *** *p* < 0.001, **** *p* < 0.0001); lethality results of initial AFB1 were compared to the degradation products of AFB1 (**C**) (* *p* < 0.05, ** *p* < 0.01, **** *p* < 0.0001). AFB1 and its degradation products induced developmental dysfunctions (**D**) in zebrafish embryos examined at 120 h. Representative phenotypic deformities induced by AFB1 were deformed tail, yolk edema, and swim bladders were not developed at 0.52, 1.77, and 4.17 nL doses; sublethal effects of the degradation products of AFB1 were not detected at either dose. Scale bar: 1 mm. The frequency of malformations of treated groups were compared to the non-injected control (non-inj-c) (Figure 1C). Quantitative data of developmental dysfunctions in zebrafish embryos are in the Supplementary Materials (Supplementary Table S1).

AFB1 treatment increased the mortality rate of 120 hpf embryos (Figure 2A). Significant decrease was detected in survival in all tested doses compared to the non-injected control (non-inj-c) (0.22 nL—* *p* < 0.05, 0.52 nL—*** *p* < 0.001, 1.77 nL—**** *p* < 0.0001, 4.17 nL—**** *p* < 0.0001). The mortality range was from 20% (±8.66%) to 52% (±2.89%) between the 0.22 nL and 4.17 nL doses. Sublethal effects of AFB1 (Figure 2D) became pronounced with increased injected dose at 120 hpf larvae. These symptoms were tail deformities, yolk edema, and swim bladders were not developed. Significant differences were observed in deformation frequencies between the non-injected control (non-inj-c) and 4.17 nL dose (* *p* < 0.05). In earlier studies, the lethal effects of AFB1 on zebrafish embryos were examined in a concentration-dependent way, which aligned with dose-dependent mortality rates in the present study [57,58]. The severity and frequency of tail deformities and yolk edema increased with dose, and the phenotypic lesions agreed with the results of Zuberi et al. [57]. In contrast, in seven days post fertilization (dpf) AFB1-treated larvae, none showed phenotypic defects in previous studies [58,59].

Degradation products of AFB1 by the NI1 strain increased the mortality of 120 hpf zebrafish embryos, but significant differences were not observed compared to the non-injected control (non-inj-c) (Figure 2B). The lethality range was from 5% (±0.00%) to 12% (±2.89%) between the lowest and highest injected volume. Malformations of degradation products were not detected at either dose (Figure 2D). Toxicological effects of degradation products have not been described earlier. Comparing the effects of AFB1 and degradation products of AFB1 on mortality (Figure 2C), the results showed that the NI1 strain was able to significantly reduce the toxic effects of initial AFB1 at all injected volumes (0.22 nL—* *p* < 0.05, 0.52 nL—* *p* < 0.05, 1.77 nL—** *p* < 0.01, 4.17 nL—**** *p* < 0.0001).

#### 2.2.2. ZEN Treatment

**Figure 3:**
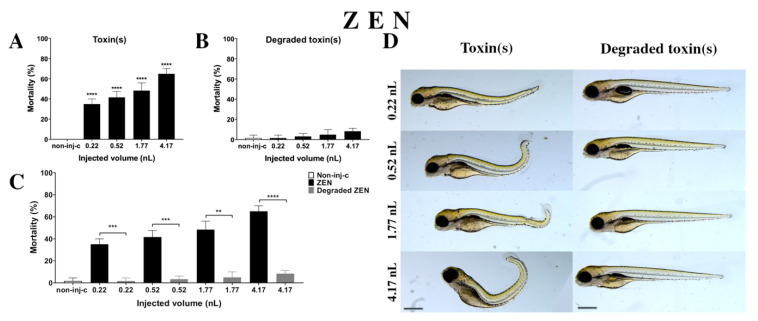
Effects of ZEN (**A**) and its degradation products (**B**) by the *Rhodococcus erythropolis* NI1 strain on 120 hpf zebrafish embryos. Mortality data are expressed as mean ± SD from three independent experiments in triplicates. Kruskal–Wallis followed by Dunn’s post-hoc test and Mann–Whitney test were used. Mortality values were compared to the non-injected control (non-inj-c) (**** *p* < 0.0001); lethality results of the initial ZEN were compared to the degradation products of ZEN (**C**) (** *p* < 0.01, *** *p* < 0.001, **** *p* < 0.0001). ZEN and its degradation products induced developmental dysfunctions (**D**) in zebrafish embryos examined at 120 h. Representative phenotypic deformities induced by ZEN were tail deformities, head and lens distortion, reduced pigmentation, and swim bladders were not developed at either dose; sublethal effects of its degradation products were not detected at either dose. Scale bar: 1 mm. Phenotypes of treated groups were compared to the non-injected control (non-inj-c) (Figure 1C). Quantitative data of developmental dysfunctions in zebrafish embryos are in the Supplementary Materials (Supplementary Table S1).

ZEN solution decreased the survival rate on 120 hpf embryos (Figure 3A). A significant increase was observed in mortality in four tested injected volumes compared to the non-injected control (non-inj-c) (0.22 nL, 0.52 nL, 1.77 nL, and 4.17 nL—**** *p* < 0.0001). The mortality ranged was from 35% (±5%) to 65% (±5%) between the lowest and highest injected volume. The severity of phenotypic defects of ZEN (Figure 3D) depended on injected dose. These malformations were tail deformities, head and lens distortions, and swim bladders were not developed. Significant increase in deformation frequencies were observed between the non-injected control (non-inj-c) and the 4.17 nL volume (** *p* < 0.01). In an earlier study, the lethal effects of ZEN on zebrafish embryos were examined in a concentration-dependent way, which aligned with dose-dependent mortality in the present study. Our results confirmed the sublethal symptoms described earlier including the curvature of body axis (dose-dependent) with abnormal heart and eye development (typical for estrogenic materials), pericardial and yolk edema, and reduced pigmentation (melanophore streak at the base of the caudal fin) [60]. However, hyperemia was not characterized in this study [61].

Degradation products of ZEN (Figure 3B) by the NI1 strain increased the mortality of 120 hpf zebrafish embryos, but significant differences were not observed compared to the non-injected control (non-inj-c). The lethality range was from 2% (±2.89%) to 8% (±2.89%) between the 0.22 and 4.17 nL injected volume. Sublethal effects of degradation products were not observed at either dose (Figure 3D). Toxicological effects of degradation products have not been described earlier. Comparing the effects of ZEN and degradation products of ZEN on mortality (Figure 3C), the results showed that the NI1 strain was able to significantly reduce the toxic effects of initial ZEN at all injected volumes (0.22 nL—*** *p* < 0.001, 0.52 nL—*** *p* < 0.001, 1.77 nL—** *p* < 0.01, 4.17 nL—**** *p* < 0.0001).

#### 2.2.3. T-2 Treatment

**Figure 4:**
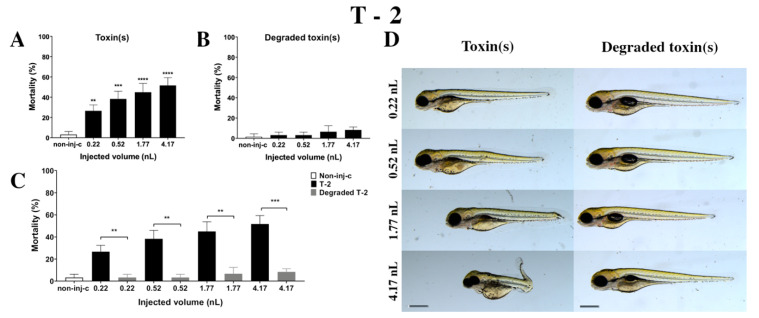
Effects of T-2 (**A**) and its degradation products (**B**) by the *Rhodococcus erythropolis* NI1 strain on 120 hpf zebrafish embryos. Mortality data are expressed as mean ± SD from three independent experiments in triplicates. Kruskal–Wallis followed by Dunn’s post-hoc test and Mann–Whitney test were used. Mortality values were compared to the non-injected control (non-inj-c) (** *p* < 0.01, *** *p* < 0.001, **** *p* < 0.0001); lethality results of initial T-2 were compared to the degradation products of T-2 (**C**) (** *p* < 0.01, *** *p* < 0.001). T-2 and its degradation products induced developmental dysfunctions (**D**) in zebrafish embryos examined at 120 h. Representative phenotypic deformities induced by T-2 were hook-like tail, tail deformities, yolk edema, head and lens distortion, and swim bladders were not developed at either dose; sublethal effects of its degradation products were not observed. Scale bar: 1 mm. Phenotypes of treated groups were compared to the non-injected control (non-inj-c) (Figure 1C). Quantitative data of developmental dysfunctions in zebrafish embryos are in the Supplementary Materials (Supplementary Table S1).

T-2 mycotoxin increased the mortality rate of 120 hpf embryos (Figure 4A). Survival decreased significantly in all injected volumes compared to the non-injected control (non-inj-c) (0.22 nL—** *p* < 0.01, 0.52 nL—*** *p* < 0.001, 1.77 nL—**** *p* < 0.0001, 4.17 nL—**** *p* < 0.0001). The lethality range was from 27% (±5.77%) to 52% (±7.64%) between 0.22 nL and 4.17 nL doses. Severity of sublethal effects of T-2 (Figure 4D) depended on the dose. Morphology changes were hook-like tail, tail deformities, pericardial edema, yolk edema, head and lens distortion, and swim bladders were not developed. A significant increase in deformation frequencies was observed between the non-injected control (non-inj-c) and 1.77 nL (* *p* < 0.05) and 4.17 nL (** *p* < 0.01). An earlier study had described the concentration-dependent mortality of T-2, which aligned with dose-dependent lethality in this study [24]. Phenotypic lesions were the same except for the lack of hatching as characterized in previous studies [24,53].

Degradation products of T-2 by the NI1 strain decreased the survival of 120 hpf zebrafish embryos, but significant differences were not observed compared to the non-injected control (non-inj-c) (Figure 4B). The mortality range was from 3% (±2.89%) to 8% (±2.89%) between the 0.22 and 4.17 nL injected doses. Sublethal effects of degradation products were not observed at any of the applied doses (Figure 4D). These results are in good agreement with a previous study, which described that NI1 strain as able to degrade the T-2 toxin for non-toxic metabolites [53]. Comparing the effects of T-2 and the degradation products of T-2 on mortality (Figure 4C), the results showed that the NI1 strain was able to reduce the toxic effects of initial T-2 significantly in all injected volumes (0.22 nL—** *p* < 0.01, 0.52 nL—** *p* < 0.01, 1.77 nL—** *p* < 0.01, 4.17 nL—*** *p* < 0.001).

#### 2.2.4. AFB1 and ZEN (AFB1+ZEN) Mixture Treatment

**Figure 5:**
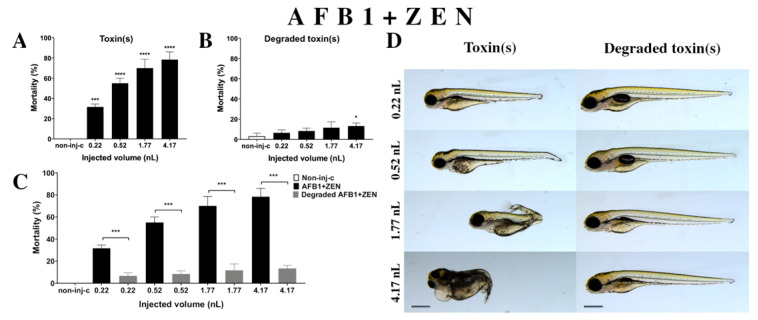
Effects of AFB1+ZEN mixture (**A**) and their degradation products (**B**) by the *Rhodococcus erythropolis* NI1 strain on 120 hpf zebrafish embryos. Mortality data are expressed as mean ± SD from three independent experiments in triplicate. Kruskal–Wallis followed by Dunn’s post-hoc test and Mann–Whitney test were used. Mortality values were compared to the non-injected control (non-inj-c) (* *p* < 0.05, **** *p* < 0.0001); lethality results of the initial AFB1+ZEN was compared to the degradation products of AFB1+ZEN (**C**) (*** *p* < 0.001). AFB1+ZEN and their degradation products induced developmental dysfunctions (**D**) in zebrafish embryos examined at 120 h. Representative phenotypic deformities induced by AFB1+ZEN were hook-like tail, tail deformities, pericardial and yolk edema, and head and lens distortion, and swim bladders were not developed at either dose; sublethal effects of their degradation products were not detected at either dose. Scale bar: 1 mm. Phenotypes of treated groups were compared to the non-injected control (non-inj-c) (Figure 1C). Quantitative data of developmental dysfunctions in zebrafish embryos are in the Supplementary Materials (Supplementary Table S1).

Combined exposure of AFB1+ZEN (Figure 5A) decreased the survival of 120 hpf zebrafish embryos. Significant increase was detected in mortality compared to the non-injected control (non-inj-c) at all injected volumes (0.22 nL—*** *p* < 0.001; 0.52, 1.77, and 4.17 nL—**** *p* < 0.0001). The mortality range was from 31% (±2.89%) to 78% (±7.64%) between the lowest and highest injected dose. Severity of malformations of AFB1+ZEN (Figure 5D) depended on the injected volume. These disorders were tail deformities, yolk and pericardial edema, head and lens distortion, and swim bladders were not developed; a significant increase in deformation frequencies was detected between the non-injected control (non-inj-c) and 4.17 nL dose (** *p* < 0.01). Our results were in good agreement with a previous study that also suggested that this mixture increased mortality depending on dose, and caused tail deformities (curvature of body axis) and yolk edema [62]. Additionally, studies have reported the toxicological effects of AFB1+ZEN mixtures in domestic animals such as feed refusal, decreased weight gain, and reduction in egg production in pig, goat, and laying hens [63,64,65]. Other studies have reported in vitro toxicological effects of mixture, where the combined effects were increased cytotoxicity in BRL 3A rat liver cells and PK15 cell line [66,67]. In addition to these, we found the seriousness of lens and head distortion and pericardial edema increased with injected volume, which had not been reported previously. In a previous report, co-occurrence of AFB1+ZEN was found in cow’s milk, core foods, composite food samples, ruminant feed, corn, maize, barley, feed and feed ingredients, sows feed, wheat, from Argentina, France, Netherlands, Turkey, Indonesia, Thailand, Brazil, Spain, United Kingdom, Germany, Czech Republic, Denmark, Portugal, and Hungary [2,68,69,70,71,72,73,74,75]. Previously, human exposure assessment had examined mycotoxins from human urine samples in different countries, where AFB1+ZEN was found in the samples from Bangladesh, Belgium, Germany, Haiti, and Spain [76,77,78,79].

Degradation products of the AFB1+ZEN mixture (Figure 5B) by the NI1 strain decreased the survival of 120 hpf zebrafish embryos, and significant differences were observed compared to the non-injected control (non-inj-c) (4.17 nL—* *p* < 0.05). The lethality range was from 7% (±2.89%) to 13% (±2.89%) between the lowest and highest injected volumes. Malformations of degradation products were not observed at either dose (Figure 5D). Toxicological effects of degradation products had not been described earlier. Comparing the effects of AFB1+ZEN and the degradation products of the AFB1+ZEN mixture on mortality (Figure 5C), the results showed that the NI1 strain was able to significantly reduce the toxic effects of initial AFB1+ZEN at all injected volumes (0.22, 0.52, 1.77, and 4.17 nL—*** *p*< 0.001).

#### 2.2.5. AFB1 and T-2 (AFB1+T-2) Mixture Treatment

**Figure 6:**
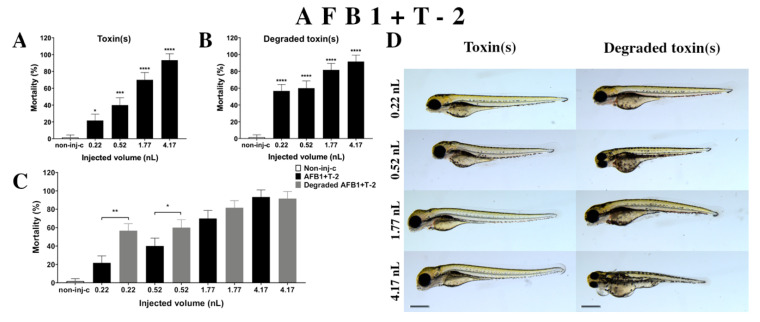
Effects of AFB1+T-2 mixture (**A**) and their degradation products (**B**) by the *Rhodococcus erythropolis* NI1 strain on 120 hpf zebrafish embryos. Mortality data are expressed as mean ± SD from three independent experiments in triplicate. Kruskal–Wallis followed by Dunn’s post-hoc test and Mann–Whitney test were used. Mortality values were compared to the non-injected control (non-inj-c) (* *p* < 0.05, *** *p* < 0.001, **** *p* < 0.0001); lethality results of the initial AFB1+T-2 were compared to the degradation products of the AFB1+T-2 mixture (**C**) (* *p* < 0.05, ** *p* < 0.01). AFB1+T-2 and their degradation products induced developmental dysfunctions (**D**) in zebrafish embryos examined at 120 h. Representative phenotypic lesions of AFB1+T-2 were tail deformities, head and lens distortion, and swim bladders were not developed at either dose; sublethal effects of their degradation products were tail deformities, pericardial and yolk edema, head and lens distortion, and swim bladders were not developed at either dose. Scale bar: 1 mm. Phenotypes of treated groups were compared to the non-injected control (non-inj-c) (Figure 1C). Quantitative data of developmental dysfunctions in zebrafish embryos are in the Supplementary Materials (Supplementary Table S1).

Combined exposure of AFB1+T-2 (Figure 6A) increased the mortality of 120 hpf zebrafish embryos depending on injected dose. A significant increase was observed in mortality compared to the non-injected control (non-inj-c) at all doses (0.22 nL—* *p* < 0.05; 0.52 nL—*** *p* < 0.001; 1.77 and 4.17 nL—**** *p* < 0.0001). The lethality range was from 22% (±7.64%) to 93% (±7.64%) between the 0.22 and 4.17 nL injected doses. Severity of sublethal effects of the AFB1+T-2 mixture (Figure 6D) depended on the injected volume. These malformations were tail deformities, head and lens distortion, and swim bladders were not developed; a significant increase in deformation frequencies was detected between the non-injected control (non-inj-c) and the highest dose (4.17 nL) (** *p* < 0.01). Studies have reported on the toxicological effects of AFB1+T-2 mixtures in domestic and lab animals such as body weight increase, oral lesions decrease, dermal lesions, hepatic injuries, bile duct proliferation, egg production decrease, feed refusal, and frequency increases in chromosomal aberrations in pigs, chickens, poultry, rat, swine, broilers, and quail [34,64,80,81,82,83,84,85,86,87]. Another study examined the in vitro toxicological effects of mixtures such as cytotoxicity in HepG2 cell line and BEAS-2B [33]. Toxicological effects of this mixture have not been reported on zebrafish embryos previously. Previously reports have analyzed co-occurrence of AFB1+T-2 in different countries such as in cow’s milk, core foods, breakfast cereals, coffee, composite samples, sow feed, wheat, maize, barley, corn, mixed feed, and silages in Argentina, France, Portugal, Spain, the Netherlands, Czech Republic, Denmark, Portugal, Hungary, and Poland [68,69,70,75,88,89,90]. Previously, human exposure assessment was examined through mycotoxins from human urine samples in different countries, and AFB1+T-2 were found in samples from Bangladesh, Belgium, Germany, Haiti, and Spain [76,77,78,79].

Degradation products of AFB1+T-2 (Figure 6B) by the NI1 strain increased the mortality of 120 hpf zebrafish embryo. Significant differences were observed between the non-injected control (non-inj-c) and all injected doses (**** *p* < 0.0001). The lethality range was from 57% (±7.64%) to 92% (±7.64%) between the lowest and highest injected doses. Phenotypic deformities induced by degradation products were observed at all injected doses (Figure 6D). These symptoms were tail deformities, yolk and pericardial edema, head and lens distortion, and swim bladders were not developed. Significant differences were observed in deformation frequencies between the non-injected control (non-inj-c) and the 1.77 and 4.17 nL doses (* *p* < 0.05). Comparing the effects of AFB1+T-2 and their degradation products on mortality (Figure 6C), the results showed that the NI1 strain was able to significantly increase the toxic effects of initial AFB1+T-2 at the two lowest doses (0.22 nL—** *p* < 0.01, 0.52 nL—* *p* < 0.05).

#### 2.2.6. ZEN and T-2 (ZEN+T-2) Mixture Treatment

**Figure 7:**
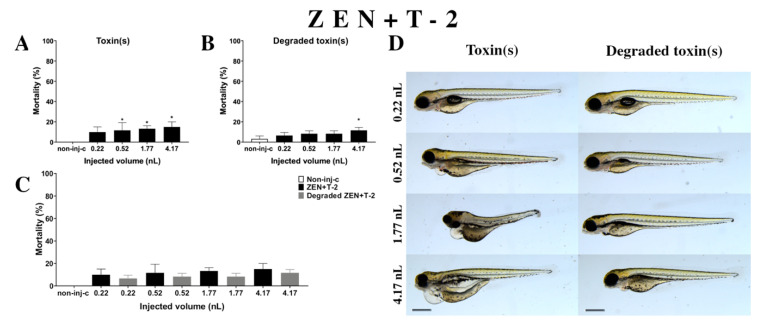
Effects of ZEN+T-2 mixture (**A**) and their degradation products (**B**) by the *Rhodococcus erythropolis* NI1 strain on 120 hpf zebrafish embryos. Mortality data are expressed as mean ± SD from three independent experiments in triplicate. Kruskal–Wallis followed by Dunn’s post-hoc test and Mann–Whitney test were used. Mortality values were compared to the non-injected control (non-inj-c) (* *p* < 0.05); lethality results of initial ZEN+T-2 were compared to the degradation products of the ZEN+T-2 mixture (**C**). ZEN+T-2 and their degradation products induced developmental dysfunctions (**D**) in zebrafish embryos examined at 120 h. Representative phenotypic lesions of ZEN+T-2 were tail deformities, pericardial and yolk edema, head and lens distortion, and swim bladders were not developed at 0.52, 1.77, and 4.17 nL; sublethal effects of their degradation products were pericardial and yolk edema, and swim bladders were not developed at 0.52, 1.77, and 4.17 nL. Scale bar: 1 mm. Phenotypes of treated groups were compared to the non-injected control (non-inj-c) (Figure 1C). Quantitative data of developmental dysfunctions in zebrafish embryos are in the Supplementary Materials (Supplementary Table S1).

Combined exposure of ZEN+T-2 (Figure 7A) decreased the survival rate of 120 hpf zebrafish embryos. Significant increase was detected in mortality compared to the non-injected control (non-inj-c) at all injected volumes except for 0.22 nL (0.52, 1.77, and 4.17 nL—* *p* < 0.05). The mortality range was from 10% (±5.00%) to 15% (±5.00%) between the 0.22 and 4.17 nL injected volume. Severity of the malformation effects of ZEN+T-2 (Figure 7D) depended on the injected volume. These disorders were tail deformities, yolk and pericardial edema, head and lens distortion, and swim bladders were not developed. Significant increase in deformation frequencies was detected between the non-injected control (non-inj-c) and 4.17 nL dose (* *p* < 0.05). Previous studies have reported the toxicological effects of ZEN+T-2 mixtures in cell line, such as cell viability decrease, ROS level increase, DNA synthesis inhibition, and myelotoxic effects in human hematopoietic progenitors, Vero cells, and the mouse fibroblast cell line L-929 [91,92,93,94]. In vivo toxicological effects of this mixture had not been reported earlier. In a previous report, co-occurrence of ZEN+T-2 was found in cow’s milk, cereals-based foods, core foods, composite food samples, feed materials, ruminant feed, maize, barley, poultry feed, sows feed, wheat, cereals, mixed feed, and silages from Argentina, Belgium, France, the Netherlands, Indonesia, Thailand, Brazil, Spain, the United Kingdom, Germany, Czech Republic, Denmark, Portugal, Turkey, Poland, Hungary, and Slovakia [2,7,68,69,70,72,73,74,75,89,90,95,96,97]. Previously, human exposure assessment examined mycotoxins from human urine samples in different countries, and ZEN+T-2 was found in samples from Bangladesh, Belgium, Cameroon, Germany, Haiti, Spain, Nigeria, and South Africa [7,76,77,78,79,98,99,100]. 

Degradation products of the ZEN+T-2 mixture (Figure 7B) by the NI1 strain decreased the survival of 120 hpf zebrafish embryos. A significant difference was observed compared to the non-injected control (non-inj-c) at the highest injected volume (4–17 nL—* *p* < 0.05). The mortality range was from 7% (±2.89%) to 12% (±2.89%) between the lowest and highest injected volume. Sublethal effects of degradation products were pericardial and yolk edema, and swim bladders were not developed (Figure 7D). Comparing the effects of the ZEN+T-2 mixture and degradation products of ZEN+T-2 on mortality (Figure 7C), the results showed that the NI1 strain was able to reduce the toxic effects of initial ZEN+T-2, but significant differences were not detected at either dose.

#### 2.2.7. AFB1 and ZEN and T-2 (AFB1+ZEN+T-2) Mixture Treatment

**Figure 8:**
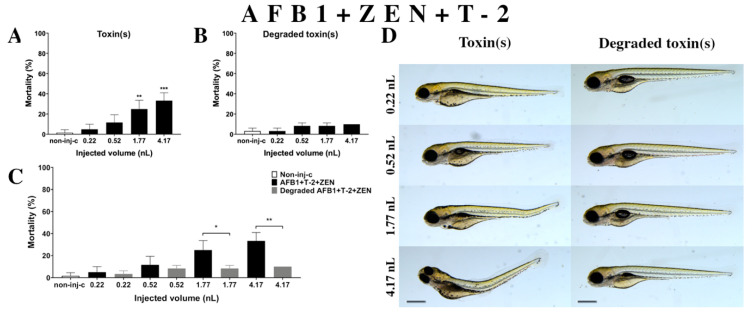
Effects of AFB1+ZEN+T-2 mixture (**A**) and their degradation products (**B**) by the *Rhodococcus erythropolis* NI1 strain on 120 hpf zebrafish embryos. Mortality data are expressed as mean ± SD from three independent experiments in triplicate. Kruskal-Wallis followed by Dunn’s post-hoc test and Mann–Whitney test were used. Mortality values were compared to the non-injected control (non-inj-c) (** *p* < 0.01, *** *p* < 0.001); lethality results of initial AFB1+ZEN+T-2 were compared to the degradation products of the AFB1+ZEN+T-2 mixture (**C**) (* *p* < 0.05, ** *p* < 0.01). AFB1+ZEN+T-2 and their degradation products induced developmental dysfunctions (**D**) in zebrafish embryos examined at 120 h. Representative phenotypic deformities induced by AFB1+ZEN+T-2 were tail deformities, yolk edema, and swim bladders were not developed at 1.77 and 4.17 nL doses; sublethal effects of their degradation products were not detected at either dose. Scale bar: 1 mm. Phenotypes of treated groups were compared to the non-injected control (non-inj-c) (Figure 1C). Quantitative data of developmental dysfunctions in zebrafish embryos are in the Supplementary Materials (Table S1).

Multiple exposure of three mycotoxins (AFB1+ZEN+T-2) (Figure 8A) increased the mortality rate of 120 hpf zebrafish embryos. A significant increase was detected in mortality compared to the non-injected control (non-inj-c) at the two highest injected volumes (1.77 nL—** *p* < 0.01, 4.17 nL—*** *p* < 0.001). The lethality range was from 5% (±5.00%) to 33% (±7.64%) between the lowest and highest injected doses. Severity of malformations of AFB1+ZEN+T-2 (Figure 8D) depended on the injected volume. These disorders were tail deformities, yolk edema, and swim bladders were not developed; however, a significant increase in deformation frequencies was not detected. Toxicological effects of this mixture had not been reported earlier. In a previous report, co-occurrence of AFB1+ZEN+T-2 was found in cow’s milk, core foods, composite food samples, feed materials, ruminant feed, maize, barley, poultry feed, sows feed, wheat, cereals, mixed feed, and silages from Argentina, France, the Netherlands, and Turkey [2,7,68,69,70]. Previously, human exposure assessment examined mycotoxins from human urine samples in different countries and AFB1+ZEN+T-2 was found in samples from Bangladesh, Belgium, Germany, Haiti, and Spain [76,77,78,79]. 

Degradation products of AFB1+ZEN+T-2 (Figure 8B) by the NI1 strain increased the mortality of 120 hpf zebrafish embryos, but significant differences were not observed compared to the non-injected control (non-inj-c). The mortality range was from 3% (±2.89%) to 10% (±0.00%) between the 0.22 and 4.17 nL injected doses. Phenotypic lesions of degradation products were not observed at any of the tested doses (Figure 8D). When comparing the effects of AFB1+ZEN+T-2 and the degradation products of the AFB1+ZEN+T-2 mixture on mortality (Figure 8C), the results showed that the NI1 strain was able to significantly reduce the toxic effects of the initial AFB1+ZEN+T-2 at the two largest doses (1.77 nL—* *p* < 0.05, 4.17 nL—** *p* < 0.01).

### 2.3. Analytical Results

Analyses of mycotoxin concentrations were performed using an ultra-high-performance liquid chromatography hyphenated with tandem mass spectrometer (UHPLC-MS/MS). The supernatants and pellets were evaluated separately. The results showed that NI1 strain was able to degrade single and combined mycotoxins in different proportions (Table 1), thus 99.69% of AFB1, 84.76% of ZEN, and 100% of T-2 was reduced in the samples, with results better than the NI1 degradation efficiency with these toxins in an earlier study (AFB1—89.35%, ZEN—60.55%, T-2—92.12%) [52]. 

Combined mycotoxins of AFB1+ZEN (AFB1: 99.82%, ZEN: 94.01%), AFB1+T-2 (AFB1: 99.84%, T-2: 100%), ZEN+T-2 (ZEN: 91.36%, T-2: 100%), and AFB1+ZEN+T-2 (AFB1: 99.84%, ZEN: 95.69%, T-2: 100%) were reduced at a higher ratio than single mycotoxins, except for the T-2 toxin. These results are in good agreement with previous study, which described that the NI strain was able to degrade the AFB1, ZEN, and T-2 higher ratio in the mixture than that of the single [52]. In addition, we found that ZEN in combination with AFB1 (94.01%) was reduced at a higher ratio than in combination with the T-2 toxin (91.36%), while the combination with the AFB1+T-2 toxin (95.69%) was reduced to the greatest extent. AFB1 in combination with T-2 and ZEN+T-2 (99.84%) was reduced at a higher ratio than in combination with ZEN (99.82%). T-2 was reduced under the detection limit regardless of the type of experiment (single or combined) and added mycotoxin (AFB1 or ZEN or AFB1+ZEN).

### 2.4. Evaluation of Interactions for Combined Mycotoxins

Several studies have described the in vitro and in vivo toxicological effects of mycotoxin mixtures with different toxicological endpoints (such as cytotoxicity, mortality). Assessing the risk of multimycotoxin exposure and hazard is difficult as different interaction effects have been reported in same mycotoxin mixtures [6,32,93,101,102].

**Figure 9:**
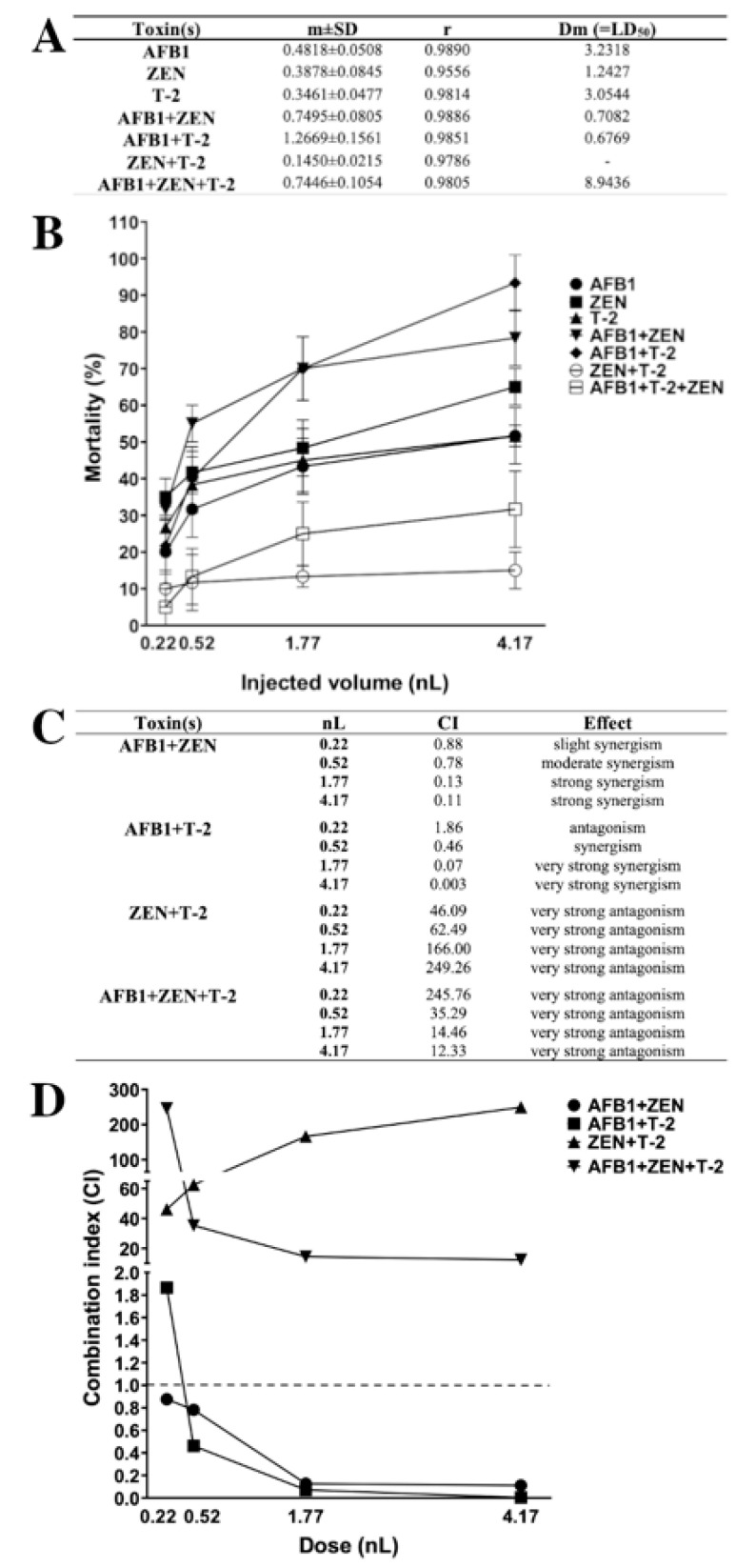
Lethal dose (LD_50_) values (**A**) and mortality curves (**B**) of mycotoxins and mixtures in zebrafish embryos (m: slope of dose-effect curve, r: compliance parameter for goodness of fit, Dm: dose that produces 50% effect, LD_50_). Type of the interaction between mycotoxins (**C**) and combination index plot of different mixtures (**D**) at each injected dose (nL). Values below and above the dashed line (CI = 1) mean synergism and antagonism. Values were calculated with CompuSyn software [105].

The combination index (CI)-isobologram is a median-effect-base method (Chou–Talalay method), which indicates the dose-response relationship, without depending on the number of substrates or products or action/inhibition mechanism [6,32,103,104,105]. This method involves plotting the dose-effect curves for each mycotoxin and their combinations. The CI value provides information on the type (antagonism, additive, synergism) and magnitude of the interaction [6]. In this study, dose-response results of individual mycotoxins and their mixtures were modeled with the median-effect equation of the mass-action law, and CI were calculated over the mortality range (Figure 9).

Lethal dose (LD_50_) values of individual and combined mycotoxins (Figure 9A) were calculated and mortality curves were plotted (Figure 9B). The results of individual exposure showed that the most toxic was ZEN (LD_50_: 1.24 nL), and the least toxic mycotoxin was AFB1 (LD_50_: 3.23 nL) on zebrafish embryos and the order of toxicity was ZEN > T-2 > AFB1. In contrast, earlier studies described that AFB1 and T-2 had higher toxic effects than ZEN on zebrafish embryos [62,106]. In combined exposure, the least toxic was ZEN+T-2 (LD_50_: not relevant), and the most toxic were the AFB1+T-2 (LD_50_: 0.68 nL) and AFB1+ZEN (LD_50_: 0.71 nL) mixtures with almost the same toxic effects on zebrafish embryos. The AFB1+ZEN mixture aligned with previously described toxicity; Zhou et al. found that AFB1+ZEN had a higher toxic effect than the other tested mixtures [62]. 

The type of interactions for combined mycotoxins were determined in CI values (Figure 9C) and CI values were plotted (Figure 9D) at each injected dose. The results showed that AFB1+ZEN synergistically enhanced the mortality of zebrafish embryos, which is in good agreement with an earlier study [62]. Additionally, previous studies have also described synergistic interactions in cell lines and domestic animals [62,65,66,67]. In addition, we found dose-dependent synergism (AFB1+ZEN mixture) on the mortality, where slight synergism was found at 0.22 nL, moderate synergism at 0.52 nL, and strong synergism at 1.77 and 4.17 nL. In the case of AFB1+T-2, we found antagonism at 0.22 nL, synergism at 0.52 nL, and very strong synergism at 1.77 and 4.17 nL on the mortality. Earlier studies that examined the interaction of AFB1+T-2 in cell lines, domestic, and lab animals found synergism at the most toxicological endpoints; antagonism was found on relative weights of liver, heart and kidney in pigs and rats [34,64,80,81,82,83,84,85,86,87]. The results showed that then ZEN+T-2 mixture had very strong antagonistic effects on the mortality in all injected doses on zebrafish embryos. In contrast, previous studies have examined the in vitro interaction of ZEN+T-2, where additivity and synergism were found at all toxicological endpoints in the cell lines [91,92,93,94]. In the case of the AFB1+ZEN+T-2 mixture, the results demonstrated that this mixture had very strong antagonistic effects on mortality in all injected volumes on zebrafish embryos. The interaction effects of mixtures, AFB1+ZEN (dose-dependent synergism) on zebrafish embryos, AFB1+T-2 (antagonism and synergism) on zebrafish embryos, ZEN+T-2 (antagonism) in vivo, AFB1+ZEN+T-2 (antagonism) in vivo, and in vitro have not been reported previously.

## 3. Discussion

The world mycotoxin survey showed that 68% of tested feed materials contained more than one mycotoxin and natural co-occurrence of mycotoxins increased health risk [107]. Co-contamination of mycotoxins confirms the elimination of mycotoxins from the food and feed chain [37,38]. Removal of mycotoxins with biological transformation may be an ideal approach, and bacterial degradation and detoxification of individual toxins have been described in several previous studies [44,45,46,47,48]. Limited data are available on the biodegradation of multiple mycotoxins, however, testing of these bacteria is important due to their increasing co-occurrence [49,50,51,52]. 

AFB1 has two main detoxification pathways: modification of difuran ring or coumarin structure. At first, the AFB1-8,9-epoxide formed, then hydrolysis resulted in dihydrodiol-derivatives. Second, the lactone ring can be changed in the coumarin moiety [49]. ZEN has two main detoxification mechanisms: both cleave a ring structure. First, hydrolysis of the ester bond in the lactone ring, followed by a spontaneous decarboxylation. Second, cleavage at the C6-ketone group resulted in lactone intermediate and subsequent activity by unspecified a/b-hydrolase, without decarboxylation [49]. The T-2 toxin detoxification pathway is de-acylation into HT-2, then T-2 triol, which is followed by de-epoxidation or de-acylation into T-2 tetraol, then de-epoxidation into de-epoxy T-2 tetraol [49]. These three mycotoxins have a few similarities: the lactone ring (aromatic ester) is the main cause of the toxicity of the AFB1 and ZEN, and carboxyl ester groups in the T-2 toxin also play an important role in the toxicity [49]. Hypothetically, this chemical structure (ester) analogue could be the common point in the biodegradation pathways at the same strain. Multiple strains of *Rhodococcus* cells can degrade both the AFB1 and T-2 toxin, and can utilize ZEN, and none can convert OTA and FB1. Therefore, these bacteria could possess active multitarget enzyme(s) detoxifying mycotoxins [108].

The detoxification efficiency of microbes is usually tested by biotests that measure only one specific effect, therefore, not suitable for testing various mycotoxin effects. Effects of AFB1 degradation products are mostly measured with SOS-Chromotest, which is a genotoxicity test. According to the earlier study by our institute, the AFB1 degradation products by NI1 are not genotoxic, and enzymes (or enzyme groups) that are responsible for the biodegradation of AFB1 are constitutive intracellular [109]. Specific enzymes of NI1 bacteria are not available as the genus of *Rhodococcus* has one hundred different aromatic ring proteases, which may be responsible for AFB1 degradation as aromatic ring degrading enzymes [109]. The effects of ZEN degradation products are commonly investigated with the BLYES test, which is an estrogenicity test. An earlier study described that ZEN degradation products by NI1 had no estrogenic effects, however, during the deeper enzymatic investigation, constitutive and indicated intracellular enzymes were not able to degrade ZEN (6-h experiment) [110]. Degrading enzymes of T-2 mycotoxins are not available. A biotest was not available to examine the effects of T-2 degradation products until the Csenki–Garai three-step method, which is an in vivo test on zebrafish embryos [53,56]. The advantages of this method are that mycotoxins and both types of metabolites (degradation products and bacterial metabolites) can be tested in a complex, synergistic and antagonistic, can be also detected, and the more efficient and safe strains can be selected.

Toxicological effects of AFB1, ZEN, T-2 in individual and combination and their degradation products were examined on zebrafish embryos in this study. Results showed that individual exposure of AFB1, ZEN, and T-2 mycotoxins significantly increased the mortality and caused different phenotypic deformities in zebrafish embryos. Based on the results of earlier published and recent studies, mycotoxins and mixtures caused the same symptoms following classical exposure and microinjection. Therefore, the microinjection-based Csenki–Garai three-step method ((1) determination of mycotoxin toxicity baseline, (2) examination of bacterial metabolites toxicity, and (3) identification of degradation products toxicity) can be used to study the effect of toxins and mixtures as the results were highly comparable with the results of classical methods. The outcomes showed that different combined exposures of AFB1, ZEN, and T-2 increased the mortality rate and caused different malformations in zebrafish embryos. The combined exposure of mycotoxins was synergistically toxic, except for ZEN+T-2 and AFB1+ZEN+T-2, which had a very strong antagonistic effect. Zhou et al. described the effects of AFB1+ZEN mixtures [62], in addition to these, we found that the seriousness of lens and head distortion and pericardial edema increased with injected volume. Toxicological effects of AFB1+T-2 on zebrafish embryos, ZEN+T-2 *in vivo*, AFB1+ZEN+T-2 mixtures *in vivo* and in vitro had not been reported in previous studies. 

Results showed that the *Rhodococcus erythropolis* NI1 strain was able to degrade mycotoxins and their mixtures to different ratios (85–100%), and mycotoxins in combination were reduced to a higher degree than single ones. The NI strain reduced the toxic effects of mycotoxins and mixtures on mortality, except for the AFB1+T-2 mixtures. Degradation products of the eAFB1+T-2 mixture by the NI1 strain were more toxic than AFB1+T-2, while the analytical results showed very high degradation, which means that the NI1 strain degraded this mixture to toxic degradation products.

The Csenki–Garai three-step method is an appropriate tool to evaluate the multimycotoxin-degrading efficiency of the *Rhodococcus erythropolis* NI1 strain and can be used for other microbial strains with similar characteristics. Limited data are available regarding the degradation and detoxification of mycotoxin mixtures, and the resulting degradation products and degradation enzymes, therefore, these should be identified in the future. The microinjection method can also be helpful in these studies by indirectly examining the effects of metabolites present in microbe degradation products.

## 4. Materials and Methods

### 4.1. Animal Protection

The Animal Protocol (2013) was approved under the Hungarian Government Regulation on animal experiments (42/2013. (II.4.)) and all studies were completed before the treated individuals reached free-feeding stage. 

### 4.2. Mycotoxin and Mixture Degradation Experiments

The *Rhodococcus erythropolis* NI1 strain (stored at −80 °C) was maintained on Luria-Bertani (LB) agar plates (10 g tryptone, 5 g yeast extract, 9 g sodium-chloride and 18 g bacteriological agar (Biolab Ltd., Budapest, Hungary) in 1 L (pH 7.0) ion-exchanged water) and incubated at 28 °C for 72 h. Then, a single colony of the strain was inoculated into 50 mL 100% LB medium (10 g tryptone, 5 g yeast extract, and 9 g sodium-chloride in 1 L (pH 7.0) ion-exchanged water) in 250 mL flasks and cultures were grown for 120 h at 28 °C, 170 rpm in a shaking incubator (Sartorius Certomat BS-1, Germany). Liquid cultures were centrifuged at 3220× *g*, 4 °C for 20 min (Eppendorf 5810R, Germany), the pellets were resuspended in 50 mL 20% sterile LB medium (100% LB medium diluted with ion-exchanged water), and then were centrifuged again in the same conditions (repeated twice). After resuspension, the optical density of the cultures was measured at 600 nm (OD600) (GENESIS 10S UV-VIS, Thermo Fischer Scientific) and adjusted to 0.6 ± 0.05 to prepare bacterial inoculum. Five mL of the bacterial suspensions were inoculated into 45 mL of sterile 20% LB medium to test the effects of bacterial metabolites. Similar inocula were prepared in parallel, which contained AFB1, ZEN, T-2 (1 mg/L final concentration (Fermentek Ltd., Israel)), and mycotoxin mixtures (1 mg/L final concentration per toxin). The microbe-free control was uninoculated 20% LB medium contaminated with AFB1, ZEN, T-2 (1 mg/L final concentration), and mycotoxin mixtures (1 mg/L final concentration per toxin). Experiments were incubated on a laboratory shaker at 28 °C, 170 rpm for 168 h in triplicate. Cultures were centrifuged at 3220× *g*, 4 °C, for 20 min. Supernatants for microinjection (1 mL) were filtered with 0.2-µm syringe filters (VWR International Ltd., Hungary) to gain bacteriologically sterile samples and stored at −20 °C. Pellets and supernatants samples were stored separately at −20 °C until analytical measurements.

### 4.3. Measurement of Mycotoxin Concentrations

UHPLC-MS/MS (ultra-high-performance liquid chromatography with a tandem mass spectrometer) was applied for the measurement of AFB1, T-2, and ZEN concentrations. First, pellets were extracted with an acetonitrile/water/formic acid (79/20/1, *v*/*v*%) mixture, then an aliquot of 500 µL extracts was taken into 1.5-mL dark vials. Supernatants in LB medium were taken directly and an aliquot of 500 µL was put into 1.5 mL dark vials. Afterward, both sample types (LB broth and pellet) were evaporated until dryness under a gentle N_2_ stream. The residues were reconstituted in 50:50 *v*/*v*% A:B mobile phases (A: water, 5 mM ammonium formate, 0.1% formic acid; B: methanol, 5 mM ammonium formate, 0.1% formic acid) and were filtered through a 0.22 µm PTFE (polytetrafluoroethylene) filter. An Agilent 1290 Infinity II UHPLC system (Agilent Technologies, USA) equipped with an Agilent Zorbax Eclipse Plus chromatographic column (2.1 × 50 mm, 1.8 μm) was used. Five μL prepared samples were injected into the mobile phase, which initially contained 95% A and 5% B eluents. Four hundred μL/min flow rate and 40 °C column temperature was set. A triple-quadruple mass spectrometer (Ultivo, Agilent Technologies, Santa Clara, CA, USA) with an ESI (electrospray) ion source was used for the determination of mycotoxin concentrations of the samples. The mass spectrometer was operated in MRM (multiple reaction monitoring) scan mode and monitored two transitions (1 qualifier, 1 quantifier) of mycotoxin precursor ions in positive ion mode. The applied analytical method was validated for LB medium. The correlation coefficient (R2) of the matrix-matched calibration was >0.9936, the recovery from LB medium spiked with the T-2 standard was 78 ± 13%, AFB1 was 114 ± 19.2% and ZEN 79 ± 4.3%, LOD (limit of detection) for T-2 was 3 μg/L, AFB1 was 0.5 μg/L, and ZEN was 0.2 μg/L. The LOQ (limit of quantification) value for T-2 was11 μg/L, AFB1 was 2 μg/L, and ZEN was 1 μg/L. 

### 4.4. Zebrafish Maintenance and Egg Collection 

Wild type laboratory-bred AB strain zebrafish were held in breeding groups of 30 females and 30 males at the Department of Aquaculture, Szent István University, Hungary, in a Tecniplast ZebTEC recirculation system (Tecniplast S.p.a., Buguggiate, Italy) at 25.5 °C ± 0.5 °C, pH 7.0 ± 0.2, conductivity 550 ± 50 µS (system water), and a light:dark period of 14 h:10 h. Fish were fed twice a day with dry granulate food (Zebrafeed 400–600 µm, Sparos Lda., Olhão, Portugal) supplemented with freshly hatched live *Artemia salina* once a day. Fish were placed in breeding tanks (Tecniplast S.p.a.) late in the afternoon the day before the experiment and allowed to spawn by removing the dividing walls the next morning. Spawning of individual pairs was delayed through time to allow a continuous supply of one-cell embryos.

### 4.5. Microinjection 

Microinjection of zebrafish embryos (microinjector, capillary puller, and parameters of capillary) was conducted as described by Csenki et al. [56]. Briefly, one-cell embryos were injected with different volumes: sphere diameter of 75 µm corresponded to an injection volume of 0.22 nL, 100 µm to 0.52 nL, 150 µm to 1.77 nL, and 200 µm to 4.17 nL. These injected volumes were selected so that the mortality values of toxins and mixtures were interpretable above and below the baseline in every dose [54]. These doses were used for each test solution (1 mg/L AFB1, ZEN, T-2, mycotoxins mixtures, bacterial metabolites, and degradation products of toxins and mixtures). After 2 h, coagulated and/or non-fertilized eggs were removed and well-divided eggs were transferred in groups of twenty into 6-cm diameter Petri dishes. Each treatment group contained 20 eggs in three replicates. Embryos were then incubated (Sanyo MIR-154) in system water at 26 °C ± 1 °C and a 14 h light and 10 h dark period and checked for lethal and sublethal effects under a microscope. System water was replaced every 24 h until 120 hpf. Digital images of larvae (120 hpf) in lateral orientation were taken under a stereomicroscope at 30× magnification (Leica M205 FA, Leica DFC 7000T camera, Leica Application Suite X, Leica Microsystems GmbH, Germany). 

### 4.6. Toxicological Endpoints

Mortality values of injected embryos were determined at 120 hpf on the basis of egg coagulation, the lack of somite formation, and the lack of heart function. Sublethal effects were examined at 120 hpf, the endpoints were hook-like tail, tail deformed, pericardial- and yolk edema, lens- and head distortion, and lack of swim bladder. The frequency of deformities was compared to the number of live embryos at 120 h. 

### 4.7. Statistics

Results were analyzed and graphs were plotted by GraphPad Prism 6.01 for Mac (GraphPad Software, San Diego, CA, USA). Data were checked for normality with the Shapiro–Wilk normality test. Significant differences were verified by Kruskal–Wallis analysis with Dunn’s multiple comparisons test and the Mann-Whitney test. Lethal and sublethal results were compared to the non-injected control (non-inj-c); mortality values of initial toxins were compared to the NI1 degradation products. CompuSyn software (Paramus, NJ, USA) was applied for the determination of interactions between mycotoxins and lethal dose (LD_50_) values [111].

## Figures and Tables

**Table 1:** Mycotoxins and their mixture degradation efficiency of the *Rhodococcus erythropolis* NI1 strain. The mycotoxin concentration of samples was detected by UHPLC-MS/MS. The supernatants and pellets of each mycotoxin and mixture were evaluated separately. The measured values of mycotoxin mixtures are listed in order of the sample labelling. Results of under detection limit are expressed as < LOD.

Samples	Before Degradation (ng ± SD)	After Degradation (ng ± SD)		Degradation Efficiency (%)
		Supernatants	Pellet	
**AFB1**	52,735 ± 4424	136.4 ± 38.2	15.07 ± 2.55	99.69
**ZEN**	24,040 ± 1164	3154 ± 105	425.7 ± 130.3	84.76
**T-2**	70,123 ± 4291	<LOD	<LOD	100.00
**AFB1+ZEN**	61,684 ± 1819	71.30 ± 4.42	13.77 ± 4.29	99.82
	29,273 ± 3301	1566 ± 99	244.7 ± 40.0	94.01
**AFB1+T-2**	52,735 ± 6326	56.58 ± 2.84	10.51 ± 2.27	99.84
	55,239 ± 6326	<LOD	<LOD	100.00
**ZEN+T-2**	21,367 ± 4699	997.3 ± 97.6	76.02 ± 64.33	91.36
	55,239 ± 3634	<LOD	<LOD	100.00
**AFB1+ZEN+T-2**	47,684 ± 2184	67.46 ± 10.55	<LOD	99.84
	23,493 ± 1938	673.5 ± 280.6	<LOD	95.69
	65,446 ± 1178	<LOD	<LOD	100.00

## Supplementary Materials

Supplementary materials can be found at https://www.mdpi.com/1422-0067/22/2/724/s1, Table S1. Effects of NI1 bacterial metabolites, single and combined mycotoxins and their NI1 degradation products on the frequency of developmental deformities (×) on 120 hpf zebrafish embryos. The frequency of deformities was determined as the number of deformed embryos (irrespective of the number of deformities per individual) compared to the number of living embryos. Frequency are expressed as mean ± SD from three independent experiments in triplicate. Kruskal–Wallis followed by Dunn’s post hoc test was used. Values were compared to the non-injected control (non-inj c) (* *p* < 0.05, ** *p* < 0.01). (td: tail and body deformities, pe: pericardial edema, ye: yolk edema, hd: head and lens distortion, sb: swim bladders were not developed).
