# Supplementary material for: Evaluation of the Multimycotoxin-Degrading Efficiency of Rhodococcus erythropolis NI1 Strain with the Three-Step Zebrafish Microinjection Method
Source: Int J Mol Sci. 2021 Jan 13;22(2):724. doi: 10.3390/ijms22020724 (PMC7828439; doi:10.3390/ijms22020724)
Supplement: Supplementary file 1 [file ijms-22-00724-s001.pdf]

**Table S1.** Effects of NI1 bacterial metabolites, single and combined mycotoxins and their NI1 degradation products on the frequency of developmental deformities (x) on 120 hpf zebrafish embryos. The frequency of deformities was determined that the number of deformed embryos (irrespective of the number of deformities per individual) was compared to the number of living embryos. Frequency are expressed as mean±SD from three independent experiments in triplicate. Kruskal-Wallis followed by Dunn's post hoc test was used. Values were compared to the non-injected control (non-inj c) (*p<0.05, **p<0.01). (td: tail and body deformities, pe: pericardial edema, ye: yolk edema, hd: head and lens distortion, sb: swim bladders were not developed; aflatoxin $B_1$ – AFB1, zearalenone – ZEN, T-2 – T-2).

| | | Toxin(s) | | | | | | Degradation products of toxin(s) | | | | | |
|---|---|---|---|---|---|---|---|---|---|---|---|---|---|
| **Toxin(s)** | | **Deformation type** | | | | | | **Deformation type** | | | | | |
| | **nL** | **td** | **pe** | **ye** | **hd** | **sb** | **Freq ±SD (%)** | **td** | **pe** | **ye** | **hd** | **sb** | **Freq ±SD (%)** |
| **AFB1** | **0.22** | x | - | x | - | x | 10.37 ± 3.39 | - | - | - | - | - | 1.75 ± 3.04 |
| | **0.52** | x | - | x | - | x | 15.16 ± 6.99 | - | - | - | - | - | 3.92 ± 3.39 |
| | **1.77** | x | - | x | - | x | 24.38 ± 19.26 | - | - | - | - | - | 5.67 ± 0.36 |
| | **4.17** | x | - | x | - | x | 28.15 ± 14.11* | - | - | - | - | - | 5.67 ± 0.36 |
| **ZEN** | **0.22** | x | - | - | x | x | 15.52 ± 7.27 | - | - | - | - | - | 0.00 ± 0.00 |
| | **0.52** | x | - | - | x | x | 19.07 ± 7.79 | - | - | - | - | - | 0.00 ± 0.00 |
| | **1.77** | x | - | - | x | x | 23.61 ± 10.48 | - | - | - | - | - | 1.85 ± 3.21 |
| | **4.17** | x | - | - | x | x | 37.11 ± 8.00** | - | - | - | - | - | 1.85 ± 3.21 |
| **T-2** | **0.22** | x | x | x | x | x | 10.52 ± 4.72 | - | - | - | - | - | 3.51 ± 3.04 |
| | **0.52** | x | x | x | x | x | 29.82 ± 9.89 | - | - | - | - | - | 3.51 ± 3.04 |
| | **1.77** | x | x | x | x | x | 88.31 ± 4.74* | - | - | - | - | - | 3.52 ± 3.06 |
| | **4.17** | x | x | x | x | x | 100.00 ±0.00** | - | - | - | - | - | 3.61 ± 3.13 |
| **AFB1+ZEN** | **0.22** | x | x | x | x | x | 7.32 ± 0.32 | - | - | - | - | - | 0.00 ± 0.00 |
| | **0.52** | x | x | x | x | x | 31.31 ± 7.22 | - | - | - | - | - | 0.00 ± 0.00 |
| | **1.77** | x | x | x | x | x | 50.00 ± 7.14 | - | - | - | - | - | 3.92 ± 3.39 |
| | **4.17** | x | x | x | x | x | 94.44 ± 9.62** | - | - | - | - | - | 3.92 ± 3.39 |
| **AFB1+T-2** | **0.22** | x | - | - | x | x | 6.42 ± 0.65 | x | x | x | x | x | 49.10 ± 9.47 |
| | **0.52** | x | - | - | x | x | 38.97 ± 0.89 | x | x | x | x | x | 55.56 ± 11.12 |
| | **1.77** | x | - | - | x | x | 43.33 ± 5.77 | x | x | x | x | x | 85.00 ± 13.23* |
| | **4.17** | x | - | - | x | x | 100.00 ± 0.00** | x | x | x | x | x | 100.00 ± 0.00* |
| **ZEN+T-2** | **0.22** | x | x | x | x | - | 1.75 ± 3.04 | - | x | x | - | - | 1.75 ± 3.04 |
| | **0.52** | x | x | x | x | x | 3.61 ± 3.13 | - | x | x | - | x | 3.61 ± 3.13 |
| | **1.77** | x | x | x | x | x | 5.67 ± 0.18 | - | x | x | - | x | 3.92 ± 3.39 |
| | **4.17** | x | x | x | x | x | 5.89 ± 0.35* | - | x | x | - | x | 3.92 ± 3.39 |
| **AFB1+ ZEN+T-2** | **0.22** | x | - | x | - | - | 9.96 ± 7.62 | - | - | - | - | - | 3.42 ± 2.96 |
| | **0.52** | x | - | x | - | - | 10.39 ± 7.63 | - | - | - | - | - | 3.71 ± 3.23 |
| | **1.77** | x | - | x | - | x | 14.21 ± 2.97 | - | - | - | - | - | 3.71 ± 3.23 |
| | **4.17** | x | - | x | - | x | 15.71 ± 4.13 | - | - | - | - | - | 5.88 ± 0.00 |
| **NI1 bacterial metabolites** | **0.22** | - | - | - | - | - | 1.67 ± 2.89 | | | | | | |
| | **0.52** | - | - | - | - | - | 1.75 ± 3.04 | | | | | | |
| | **1.77** | - | - | - | - | - | 3.71 ± 3.23 | | | | | | |
| | **4.17** | - | - | - | - | - | 3.71 ± 3.23 | | | | | | |
| **Non-inj-c** | **-** | - | - | - | - | - | 0.00 ± 0.00 | | | | | | |
